# The impact of brain-derived neurotrophic factor gene polymorphisms on post-stroke naming in aphasia

**DOI:** 10.1371/journal.pone.0327320

**Published:** 2025-07-14

**Authors:** Matilda Randighieri, Alyssa Devine, Lindsey Kelly, Victoria Tilton-Bolowsky, Voss Neal, Joseph Kang, Julian Bösel, Argye Elizabeth Hillis, Melissa D. Stockbridge

**Affiliations:** 1 Department of Neurology, Johns Hopkins University School of Medicine, Baltimore, Maryland, United States of America; 2 Medical Faculty – Mannheim, University of Heidelberg, Mannheim, Germany; 3 Medical Faculty – Heidelberg, University of Heidelberg, Heidelberg, Germany; 4 Department of Neurology, University Hospital Heidelberg, Heidelberg, Germany; Nathan S Kline Institute, UNITED STATES OF AMERICA

## Abstract

Post-stroke aphasia, or language deficits after stroke, afflicts 20–30% of survivors and often persists into the chronic phase. The protein brain-derived neurotrophic factor has been identified as important for neuroplasticity, and is regulated by the brain-derived neurotrophic factor gene. A patient’s brain-derived neurotrophic factor genotype may influence their post-stroke aphasia recovery. This study aimed to investigate the impact of a single nucleotide polymorphism in the brain-derived neurotrophic factor gene, rs6265, on language recovery. We hypothesized that individuals with the most common polymorphism would exhibit better chronic naming performance and a more favorable recovery trajectory from poor acute performance to strong chronic outcomes compared to those without the polymorphism. We retrospectively analyzed data from 77 participants with post-stroke aphasia from three recent or ongoing studies that included both repeated standardized picture naming assessments in the acute, subacute, and chronic phases and brain-derived neurotrophic factor genotyping. Statistical analyses controlled for acute performance and lesion volume when evaluating the effect of brain-derived neurotrophic factor genotype on the probability of better chronic language recovery (Aim 1) and on the probability of a person with poor acute performance later having strong performance in the subacute to chronic period (Aim 2). Results indicated that those with the most common polymorphism had a 33% higher likelihood of high naming scores in the chronic phase compared to those with the with less common polymorphisms (with a methionine allele). Individuals with the typical polymorphism whose acute naming was below average after stroke exhibited a 24% higher likelihood of recovering to be above average. Brain-derived neurotrophic factor status was not a significant independent predictor of outcome in either model. Our results suggest that the effect of brain-derived neurotrophic factor polymorphisms on chronic post-stroke aphasia recovery is, at best, modest and underscores the importance of individualized approaches to neurorehabilitation.

## Introduction

Despite significant advancements in the treatment of ischemic stroke, post-stroke language impairment, or aphasia, affects nearly one in three survivors [1]*.* The exact mechanisms underlying recovery have yet to be fully characterized, but are thought to progress from acute restoration of blood flow to ischemic tissue [2–4] to upregulation of the distributed right-hemisphere language network homologues in the subacute period [5–7]. Later functional improvement is supported by compensatory reorganization of preserved language networks [8]. Thus, neuroplasticity is a key aspect of recovery, and brain-derived neurotrophic factor (BDNF) is one mediator of post-stroke aphasia recovery that has gained attention in recent years [9–13].

First isolated in 1982, BDNF is the most abundant neurotrophin in the adult brain. It plays a fundamental role in the maintenance of central nervous system development and functioning, and its dysfunction has been linked to the progression of neurological diseases [14]. Physiologically, BDNF regulates neuronal differentiation, development, and survival; it fosters synaptic transmission and activity-dependent plasticity [14–17]. Unlike most other growth factors, secretion of BDNF is both constitutive and activity-dependent in response to neuronal activity [18,19]. Activity-dependent BDNF release in particular plays a key role in mediating and promoting synaptic plasticity in cortical cells, hippocampal cells and beyond [20], which indicates that BDNF could be a key factor in learning and memory functions involved in stroke pathophysiology and prognosis. Animal studies demonstrate a neuroprotective effect of intravenous BDNF–gene-modified human mesenchymal stem cells injection in reducing infarct size [21,22], as well as intraventricular BDNF pretreatment preserving pyramidal cell integrity in the hippocampus post-ischemia [23].

The BDNF *Met* allele variant is a naturally occuring single nucleotide polymorphism (SNP) which involves a substitution of valine to methionine at codon 66 (*Val*^*66*^*Met* or *Met*^*66*^*Met,* instead of *Val*^*66*^*Val*). The *Met* allele is associated with impairment in the activity-mediated secretion of BDNF, resulting in 18%–30% (*Val/Met*: 18%; *Met/Met*: 30%) less activity-dependent secretion [24,25]. The *Met* allele also has been associated with poorer memory, decreased learning and cortical plasticity, smaller brain activation volume, and other indicators of poor function [26–31], while non-carriers (those with the typical polymorphism, *Val/Val*) show greater functional brain activation [32]. Early studies demonstrated that carriers of the *Met* allele experience increased stroke risk, poorer stroke outcomes, and poorer recovery [33–35]. However, more recent studies have suggested a more nuanced and complex interaction between the *Met* allele and stroke pathophysiology [18].

### *Met* allele and aphasia recovery

A recent systematic review of studies examining the role of BDNF in post-stroke aphasia recovery in the last 20 years [13] identified only three studies: de Boer et al. [11], Fridriksson et al. [10] and Kristinsson et al. [12] focused on this BDNF single nucleotide polymorphism. De Boer et al. included participants in the acute to subacute phase of recovery (first three months), while Fridriksson et al. and Kristinsson et al. included those in the chronic phase (per the authors, 65 of the 87 participants described in Kristinsson et al. previously had been described in Fridriksson et al.). No significant differences in language improvement were found between noncarriers and carriers of the *Met* allele who received aphasia-focused speech-language therapy throughout the acute and subacute period [11] on either an assessment of communication in daily life situations (Amsterdam Nijmegen Everyday Language Test) or the 60-item Boston Naming Test, a picture naming test of nouns [36]. The patients with chronic post-stroke aphasia described by Kristinsson et al. [12] and Fridriksson et al. [10] received anodal transcranial direct current stimulation with speech-language therapy, an intervention that is thought to enhance neural plasticity through a BDNF-dependent mechanism [37]. In these investigations, *Met* allele carriers experienced more severe language impairment at baseline and poorer recovery than those with the *Val/Val* polymorphism. Those with the typical polymorphism also showed significantly greater task-specific brain activation in both hemispheres. The apparent discrepancy between the limited available acute and chronic studies’ findings may be due to the fact that effects of the *Met* allele are subtle relative to the high variability in early stroke recovery, characterized by a reduction of 18%–30% in activity-dependent BDNF secretion. De Boer et al. speculated that unexpectedly high variability among patients in the early post-stroke period relative to sample size (*n *= 53) and the decision not to account for stroke severity in analysis may have influenced their findings. It is also possible that effects of differences in BDNF secretion might accumulate gradually, showing only when long-term aphasia recovery in the chronic phase is observed [38].

### Summary and present work

The BDNF genotype is being increasingly investigated as potential valuable biomarker in post-stroke recovery across domains of function. However, the prior work in aphasia has been considerably limited by low sample sizes and repeated reporting of the same subgroup of patients. The studies of BDNF in aphasia also differ in other methodological ways that make it difficult to draw clear comparison. Small sample sizes are a particularly critical issue in this case, as the prevalence of the *Met* allele in the US population is low. Moreover, the evidence from investigations into the effects of BDNF genotype is mixed. It is very possible that BDNF status influences different phases of recovery in different ways. New knowledge is to be gained by examining post-stroke phases of recovery with improved granularity in the context of BDNF and communication. These are limitations that the present work aims to address with a sample size.

To our knowledge, no previous study systematically examined both the influence of BDNF genotype on chronic language outcomes and on recovery trajectories across different timepoints. We argue that following the same cohort across the different stages of the recovery process is essential to detect potential genotype-related differences in recovery trajectory.

Also, assuming that cumulative effects of (altered or physiological) BDNF secretion may become more evident in the chronic phase, a longitudinal design is needed. This study also has a significant clinical relevance: identifying genetic predictors such as BDNF polymorphism could help clinicians to identify patients with different recovery potential early on, basing among others on their genetic profile, and treat them accordingly, offering tailored rehabilitation and potentially better long-term outcomes.

In this investigation we examined two questions related to aphasia recovery and BDNF genotype. First, we examined whether *Val/Val* BDNF genotype had a positive effect on the probability of better chronic language performance. We predicted that people with *Val/Val* genotype would have a greater probability of higher performance in naming during the chronic phase. Our second question related to trajectory of performance. We examined whether typical BDNF genotype (*Val/Val*) had a positive effect on the probability of a person with poor acute performance later having strong performance in the subacute to chronic period, and we predicted that having a *Val/Val* genotype may improve the chances of a better later performance among participants whose acute performance was poor.

## Materials and methods

### Participants

**Fig 1:**
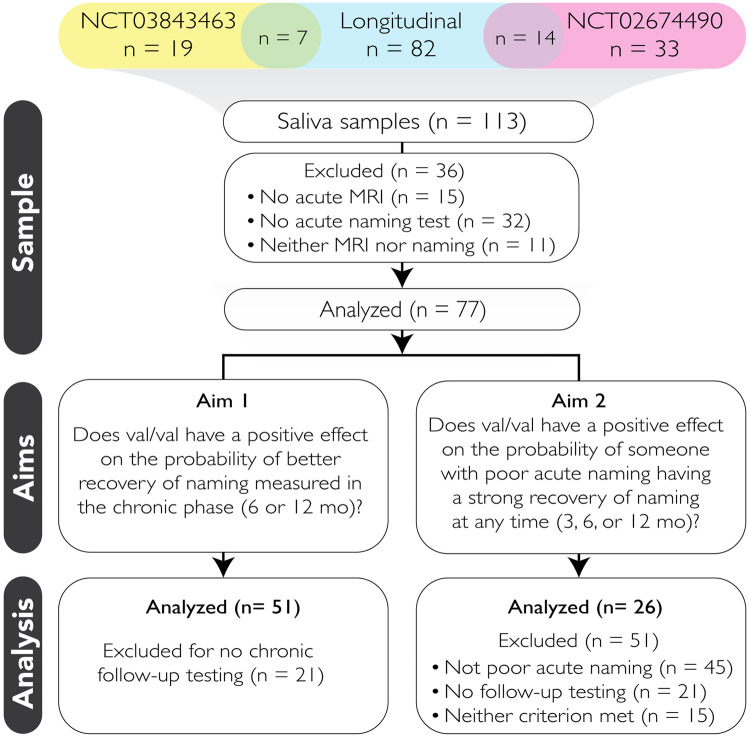
Sample Characteristics. Across three studies, 113 patients had provided saliva samples. Of these, 77 had adequate behavioral data to be analyzed pursuant to at least one of the two study aims.

This research is based on the retrospective analysis of prospectively collected data from 113 participants with post-stroke aphasia, drawn from three different research studies from our group: two clinical trials for the treatment of aphasia and one longitudinal observational study (Fig 1). Each of the three studies employs distinct methodologies tailored to its research aims, with some common aspects in their aphasia-related data acquisition and inclusion/exclusion criteria. SLISSE (Stroke Language Intervention Studies for Subacute Aphasia; NCT02674490) was a randomized controlled trial (RCT) focused on evaluating the effects of A-tDCS neurostimulation (versus sham) with language intervention for subacute post-stroke aphasia [39]. ELISA (Escitalopram and Language Intervention for Subacute Aphasia; NCT03843463) is an RCT that investigates the potential benefits of escitalopram as an adjunct to language intervention in subacute aphasia [40]. The longitudinal observational study tracks the progression of communication-related stroke recovery over time, providing insights into recovery-relevant stroke neuroimaging and prognostic indicators. ELISA is a multisite RCT; the other studies recruited from the Johns Hopkins Hospital or Johns Hopkins affiliated hospitals. Participants included 33 recruited from SLISSE, 19 from ELISA, and 82 from the observational, longitudinal study. Dual enrollment with an RCT and the observational study was common: 14 participants were in both SLISSE and the observational study, 7 in both ELISA and the observational study, for a total of 113 participants.

Across the three studies, participants had to be 18 years of age or older and pre-morbidly fluent in English by self-report. Each participant provided written consent for inclusion. Participants with a history of major psychiatric disease or neurological disease affecting the brain (other than stroke) were excluded. Further specific inclusion criteria for each study were as follows. Patients in SLISSE were consented within 90 days of left hemisphere stroke onset and were required to have an aphasia diagnosis (Aphasia Quotient ≤ 93.7 on the Western Aphasia Battery-Revised, WAB-R [41]) and be right-handed at baseline. Patients in ELISA are consented within 7 days of onset and must have aphasia, but the study permits left-handed individuals. The observational study is the most inclusive, as it does not require patients to have an aphasia diagnosis; however, patients must consent within the first 5 days of either left-hemisphere stroke onset or extubation. Participant characteristics for the present analysis are summarized in Table 1. No significant differences were observed between the two groups (*Val/Val*, ≥ 1 *Met*) within the subgroup analyzed for either Aim 1 or Aim 2.

**Table 1:** Characteristics of the analyzed samples.

		Aim 1		Aim 2	
		*Val/Val* *n *= 36	≥1 *Met* *n *= 15	*Val/Val* *n* = 19	≥1 *Met* *n* = 7
F:M (*n*)		13:23	9:6	11:8	5:2
Age (years)		62(12)	59(14)	62(15)	57(19)
Race & Ethnicity, *n*	White	11	8	3	4
	Black	21	6	14	3
	Asian	4	1	2	0
	Hispanic	0	1	0	1
	Non-Hispanic	36	14	19	6
Education (years)		14(3)	15(2)	13(2)	14(2)
Lesion Vol (cc)		29(61)	10(15)	52(78)	19(19)
Acute testing DPO		4(3)	3(3)	3(2)	4(3)
NIH Stroke Scale		7(6)	6(6)	10(7)	9(8)
Aphasia severity		68(38)	67(33)	44(37)	38(23)
Global		7	2	7	2
Wernicke’s		4	1	3	1
Broca’s		1	4	1	4
Transcortical motor		1	0	1	0
Anomic		8	4	6	0
None		14	3	1	0
Naming	Acute	−0.2(1.1)	−0.1(0.9)	−1.2(0.7)	−1.0(0.7)
	3 months	0.3(1.0)	0.6(0.8)	−0.3(1.0)	0.1(1.0)
	6 months	0.3(0.9)	0.4(1.0)	−0.4(1.0)	−0.1(1.2)
	12 months	0.2(1.0)	0.3(1.0)	−0.5(0.9)	−0.9(1.4)

Results are reported as Mean (Standard Deviation) unless otherwise noted. Within each aim, groups were statistically similar in every measure. DPO: Days post onset. Aphasia severity and subtype were based on performance on the Western Aphasia Battery – Revised. Naming accuracy was based on performance on the Boston Naming Test or Philadelphia Naming Test. Participants included in Aim 1 analyses had to have completed follow-up evaluation during at least one chronic timepoint. Participants included in Aim 2 analyses had to have Completed follow-up testing at any timepoint. Where testing from multiple follow-up timepoints was available for an individual, these were averaged prior to analyses to avoid disproportionate statistical influence.

Data were accessed on Nov. 6, 2023 for research purposes. Authors had access to information that could identify individual participants during the data review.

### Behavioral measures

All participants received the Western Aphasia Battery-Revised, WAB-R [41] at baseline. This battery is widely used to characterize language after stroke and includes subtests that evaluate four domains: discourse when describing a picture, auditory verbal comprehension, repetition (up to 20 syllables), and naming and word finding. Auditory verbal comprehension is assessed in three parts. First, patients are asked simple and complex yes/no questions. Next, patients must indicate the correct object or pictured item, shape, letter, number, color, body part, or piece of furniture in the room when the target’s label is provided. In order to receive full points, patients must be able to correctly indicate left versus right sided body parts. Finally, patients must follow simple and syntactically complex sequential commands using provided objects. Naming and word finding also are assessed using multiple separate tasks. Patients must name a series of common objects, demonstrate category fluency (name as many animals as they can in a minute), complete sentence starters appropriately, and respond to questions of general knowledge (e.g., “How many days are in a week?”). Aphasia subtypes are determined on the basis of relative strengths and weaknesses on the battery. Those with global aphasia have the most severe impairment across all four domains. Wernicke’s aphasia is associated with relatively preserved discourse fluency in the presence of relatively poor comprehension, repetition, and naming and word finding. Broca’s aphasia is associated with relatively preserved comprehension in the presence of relatively poor fluency, repetition, and naming and word finding. Transcortical motor aphasia is associated with relatively preserved comprehension and repetition in the presence of relatively poor fluency and naming and word finding. Anomic aphasia is associated with minimal impairment of fluency, comprehension, or repetition, in the presence of persisting difficulties with naming and word finding. This is considered the mildest form of recognized aphasia on the battery. Finally those with no aphasia per the Western Aphasia Battery have achieved at least 93.7% of the total points possible, though subtle persisting deficits, particularly in complex and conversational discourse commonly are reported by these individuals [42].

All participants included in the analysis had data from at least two of the following time points: (1) acute, within 5 days post-onset or extubation; (2) early subacute, 15–90 days post-onset; (3) late subacute, 120–180 days post-onset; and (4) chronic, 210–365 days post-onset. Each study used a comparable picture naming assessment targeting familiar nouns. The Boston Naming Test 30-item version [43] was used at the first timepoint in the SLISSE trial and at each of the four timepoints in the longitudinal trial. The 60-item version [36] is used in the first timepoint in the ELISA trial. The Philadelphia Naming Test (PNT) 175-item version [44] was used in every assessment in SLISSE trial and the 30-item version [45] is used in every assessment in the ELISA trial. In SLISSE, the 175-item PNT was administered twice on subsequent days at each timepoint and performance is averaged to increase the stability of the measure. In ELISA, the shorter 30-item PNT’s two non-overlapping, counterbalanced forms (A and B) are administered in a single session, and the performance on these is averaged. In order to analyze naming performance for the present investigation, each test was separately z-transformed, then the average z-score across naming measures for a given timepoint was used as the dependent variable for that timepoint.

### Neuroimaging data

Participants underwent diffusion-weighted imaging (DWI) and/or FLAIR MRI scans as part of routine acute clinical care and/or pre-treatment assessment. Protocols and clinical imaging equipment varied among hospitals. Subacute (pre-treatment) FLAIR imaging was performed using a 3T Philips Achieva or Elition RX scanner (Philips Healthcare, Best, The Netherlands) with the following parameters: TR, 11,000 ms; TE, 100 ms; 2 mm slice thickness (69 slices). Lesions were manually traced by hand by trained research group members on either acute DWI or subacute FLAIR using using MRIcron (available at nitrc.org). Anatomical images and lesion masks were normalized to MNI space using Statistical Parameter Mapping (SPM-12) routines for DWI and ClinicalToolbox for FLAIR. Lesion volumes were then extracted using MRIcron.

### BDNF genotyping

*Val/Met* and *Met/Met* (*rs6265* or *G196A*) was detected via saliva sample using Genotek Oragene Discover OGR-500 and OGR-575 kits. OGR-500 kits were utilized for patients able to provide a 2 mL saliva sample independently resulting in a median yield of 110 µg. OGR-575 kits include a sponge for assisted collection of 0.75 mL saliva from patients unable to provide a sample independently and have a median yield of 17.3 µg. Each sample was labeled with the participant’s de-identified study number and stored at room temperature per manufacturer’s instructions. All samples were sent to the Johns Hopkins Medicine Genetic Resources Core Facility, where DNA was isolated using the Revvity Magnetic Separation Module 1 and the chemagic DNA Blood 10k Kit (CMG-704). The samples were quantified on the Nanodrop ND-1000 Spectrophotometer and normalized to 10ng/ul. 30ng of DNA was used in the PCR. The rs6265 primer and the TaqPath ProAmp Master Mix (A30865) were purchased from Thermo Fisher. End-point PCR and analysis were performed on the Applied Biosystems 7900HT Fast Real-Time PCR System using the Sequence Detection System Software.

### Statistical analysis

The available data were limited by two potentially influential confounding factors, which we designed our statistical plan to mitigate. First, relatively few people in the sample demonstrated the *Val/Met* or *Met/Met* genotype. Second, we realized that, although differences in lesion volume were not significant, those with the *Met* allele had smaller lesions, and there was less variability in lesion size than in the larger *Val/Val* group. In order to address both issues while maximizing the available data to be analyzed, we chose to evaluate the probability of categorically coded outcomes in binary logistic regressions that controlled for acute linguistic performance and lesion volume. This approach also sought to address the methodological weaknesses identified by de Boer et al. in the only prior investigation of the effect of BDNF genotype on early aphasia recovery in the literature, that variability among patients in this period was greater than anticipated and their statistical approach had not accounted for stroke severity.

In examining the first aim, the binary logistic regression examined whether dominant BDNF status (*Val/Val*) had an effect on the probability of better or worse chronic language performance. Performance was characterized based on whether the z-transformed naming score when measured 6 or 12 months after stroke was positive or negative.

To evaluate the second aim, we examined whether dominant BDNF status (*Val/Val*) had an effect on the probability of a person with poor acute performance later having strong performance, operationalized as someone whose z-transformed naming score switched from negative during the acute phase to positive when measured at any other timepoint (3, 6, or 12 months after stroke). A total of 26 patients had negative z-transformed acute naming performance that could be compared to at least one follow-up assessment. Our prediction was that *Val/Val* would be associated with a positive odds ratio in both Aim 1 and Aim 2. Although we did not have specific predictions about the models as a whole, or significance testing associated with any independent predictor, we have reported on the models in full.

Requests for the complete deidentified dataset may be made to Johns Hopkins University School of Medicine Institutional Review Board (jhmeirb@jhmi.edu) resulting in a formal data sharing agreement.

## Results

### Aim 1: Does *Val/Val* BDNF status have an effect on the probability of good chronic naming performance in people with aphasia?

In a model predicting the likelihood of above-average chronic naming performance, acute performance, lesion volume, and BDNF status explained 71% of the variance in goodness of chronic performance (Nagelkerke’s *R*^2^ = 0.71), and the overall model was significant, χ^2^(3) = 32.41, *p* < 0.001. Holding acute naming and total lesion volume constant, the odds of a positive z-transformed naming performance any time after 6 months for those with *Val/Val* over the odds of a positive z-score on naming any time after 6 months for those with the *Met* allele is 1.33 (odds ratio) or 33% higher for those with *Val/Val* than *Val/Met*. BDNF status was not a significant independent predictor (Table 2). Only acute naming performance was a significant independent predictor of the probability of later above-average naming performance.

**Table 2:** Logistic regression predicting good naming performance in chronic phase.

	B	S.E.	Wald	Sig.	Odds Ratio [95% C.I.]
Constant	2.83	1.05	7.23	0.01	16.97
Acute naming	2.33	0.74	9.80	0.002	10.26 [2.39, 44.05]
Lesion volume	−0.01	0.01	0.47	0.49	0.99 [0.97, 1.02]
BDNF	0.28	1.16	0.06	0.81	1.33 [0.14, 12.97]

### Aim 2: Does *Val/Val* BDNF status have an effect on the likelihood that people with poor acute performance later perform well?

When considering only those whose acute performance was below average, a model predicting who among them would go on to have above average naming performance from acute performance, lesion volume, and BDNF status explained 39% of the variance (Nagelkerke’s *R*^2^ = 0.39), a significant overall model (χ^2^(3) = 8.99, *p* = 0.03). The odds of *switching* from a negative z-score in acute performance to a positive z-score in follow up at any point for those with *Val/Val* over the odds of positive z-score in follow up at any point for those with the *Met* allele is 1.24 (odds ratio) or 24% higher for those with *Val/Val* than *Val/Met*, although this difference was not statistically significant (Table 3). Only acute naming performance approached significance as an independent predictor.

**Table 3:** Logistic regression predicting positive recovery.

	B	S.E.	Wald	Sig.	Odds Ratio [95% C.I.]
Constant	1.97	1.12	3.11	0.08	7.19
Acute naming	1.51	0.78	3.75	0.05	4.53 [0.98, 20.92]
Lesion Volume	−0.01	0.01	0.95	0.33	0.99 [0.97, 1.01]
BDNF	0.22	1.04	0.04	0.83	1.24 [0.16, 9.56]

## Discussion

This study aimed to investigate the influence of the BDNF genotype on aphasia recovery following a stroke. First, we examined whether BDNF genotype had an effect on the probability of better chronic language performance. Second, we examined whether typical BDNF genotype had an effect on the probability of a person with poor acute performance later having strong performance.

Our findings pursuant to the first aim showed that BDNF *Val/Val* genotype was associated with a positive odds ratio. However, our results did not indicate that the BDNF *Val/Val* genotype was associated with a significant effect on the probability of better chronic language recovery, as the participants with the *Val/Val* genotype had only a 33% higher likelihood of achieving above average naming scores in the chronic phase compared to those with the *Val/Met* genotype. This finding was broadly consistent with the only similar prior investigation into aphasia recovery investigated in this timeframe—de Boer et al. [11], who also did not observe a significant effect of BDNF on aphasia outcomes. Although we analyzed a similarly sized sample of participants, we sought to address the limitations the authors identified directly both by employing an *a priori* categorical analysis strategy and by accounting for acute stroke severity statistically in the logistic regression. We also utilized a broader range of picture naming assessments, many of which were administered twice at each timepoint to improve stability. Thus, these findings strengthen one another in evidencing that BDNF polymorphisms are not a substantial driver of aphasia recovery in early stroke, though we provide evidence that they constitute a modest positive influence.

A limitation of our examination of Aim 1 was the high incidence of relatively mild language impairments within the sample. All participants who otherwise qualified were included in analysis, including 33% (17/51) of participants included in Aim 1 who did not meet the WAB-R criteria for a diagnosis of aphasia. As all of the naming assessments typically used in the evaluation people with aphasia are designed for the detection of impairment, items skew toward greater frequency in English usage and earlier age of acquisition, making them easier items to name. This also can contribute to a scale attenuation or “ceiling effect” when measuring performance in patients whose impairments are subtle.

Aim 2 was designed to complement Aim 1 and mitigate this limitation. When only those whose initial naming after stroke was poor were included in analysis, we again observed that BDNF *Val/Val* genotype was associated with a positive odds ratio of recovering to an above average performance in naming. The *Val/Val* genotype was associated with a 24% higher likelihood of switching from a negative to a positive z-transformed naming score over time. As in Aim 1, however, this was not a significant independent predictor of later naming performance within the model. Unexpectedly, the odds ratio in this case was of a slightly smaller magnitude than the odds ratio in Aim 1 (33%).

Recent reviews [13,38] have concurred that only four prior studies have directly examined the effect of any genetic variant on aphasia recovery, and all four have examined BDNF rs6265 [10–12,37]. Two studies in acute to subacute recovery have yielded null results, while two studies in chronic recovery have rejected the null. In each phase of care, one of the two studies has involved the use of non-invasive brain stimulation [10,37]. Even when significant effects are associated with BDNF polymorphisms, the effect size is small, and it is possible that it may only accumulate to detectable levels over an expanded timeline of recovery [38]. Our findings contribute to this emerging body of work.

### Limitations and future directions

While small sample sizes constitute a clear limitation in this and many prior investigations of aphasia recovery beginning in the acute phase, another major issue in studies examining BDNF polymorphisms, as also emphasized by Mayes et al. [46], is the uneven distribution across populations and underrepresentation, at least in some samples, of atypical BDNF genotypes, particularly *Met/Met* individuals. Our study is subject to this limitation as well, as only a small proportion of our participants carried the *Met* allele. In the US, the *Met* allele has a prevalence in the US of 27% for heterozygotes and 5% for the homozygotes [25,47] and varies significantly across racial and ethnic groups. It is present in 30% in of people identified as Caucasian/White, 70% in people of Asian descent [48] and is almost absent in African American/Black and Native American populations [49]. Given that Baltimore City, Maryland (the primary recruitment site for this study) is approximately 60% Black or African American, 27% White, and 2.5% Asian, it is unsurprising that our sample, reflecting this variability, contained relatively few *Met* allele carriers. The demographic context is therefore crucial when interpreting our genetic findings, as it likely contributed to reduce statistical power to detect genotype-based differences. But the implications of this limitation extend beyond power considerations. Subramanian et al. [50] recently emphasized the role of ethnicity in modulating stroke recovery outcomes, particularly in the motor domain. Although our study was not statistically powered to conduct ethnicity-stratified analyses, future research that is deliberately powered for these analyses will enable rigorous investigation of potential gene–ethnicity as well as gene–environment interactions in post-stroke recovery. If replicated and better characterized, these interactions could support the development of new rehabilitation approaches that account for both neurobiological and social or ethnical determinants of recovery. In fact, if and when we know about differences in potential poorer outcomes based on ethnicity, we have the ability to shift our clinical monitoring and treatment to respond to that vulnerability and provide equitable and necessary support for all individuals.

Another limitation is that patients with more severe disabilities and outcomes are disproportionately lost to follow-up in longitudinal designs, due to the burden of ongoing participation. This may have prevented us from seeing a more evident impact on recovery. We did not control for participants’ access to standard aphasia treatments or participation in treatment-specific research studies, which may have influenced recovery outcomes, as many patients in our community do not have access to rehabilitation services after stroke.

Our main objective was to determine whether, after controlling for lesion volume and acute performance, the *Val/Val* genotype positively affected the chances of a good recovery, for which we could not obtain sufficient evidence. Future research should consider strategies for enriched sampling of participants with the target BDNF polymorphism and do so in larger trials. Investigating the interactions between BDNF genotypes and specific rehabilitation approaches and adjuvants, such as non-invasive brain stimulation could provide further insights into personalized and optimized post-stroke aphasia treatment.
